# Broad Electrocardiogram Syndromes Spectrum: From Common Emergencies to Particular Electrical Heart Disorders

**DOI:** 10.3390/jpm12111754

**Published:** 2022-10-22

**Authors:** Anastasia Balta, Alexandr Ceasovschih, Victorița Șorodoc, Kyriakos Dimitriadis, Sara Güzel, Cătălina Lionte, Cristian Stătescu, Radu Andy Sascău, Emmanouil Mantzouranis, Athanasios Sakalidis, Panayotis K. Vlachakis, Panagiotis Tsioufis, Athanasios Kordalis, Eleftherios Tsiamis, Konstantinos Tsioufis, Laurențiu Șorodoc

**Affiliations:** 1 Faculty of Medicine, University of Medicine and Pharmacy Grigore T. Popa, 700115 Iasi, Romania; 2 2nd Internal Medicine Department, Sf. Spiridon Clinical Emergency Hospital, 700111 Iasi, Romania; 3 1st Cardiology Clinic, Medical School, National and Kapodistrian University of Athens, Hippokration Hospital, 11527 Athens, Greece; 4 Cardiology Department, Cardiovascular Diseases Institute, 700503 Iasi, Romania

**Keywords:** electrocardiogram, Wellens’s syndrome, De Winter syndrome, Kounis syndrome, Takotsubo (Broken Heart) syndrome, Yamaguchi syndrome, WPW syndrome, LGL syndrome, Brugada syndrome, PAFIYAMA syndrome, Haissaguerre syndrome, Osborn syndrome, LEOPARD syndrome

## Abstract

Electrocardiogram (ECG) still remains a very useful diagnostic method in modern cardiology. Its broad availability, noninvasiveness and good sensitivity explain why it plays a capital role in the very beginning of the process of diagnosis for every patient, with or without cardiac-related complaints. For the practitioner, good training in ECG interpretation is mandatory. Sometimes, the ECG trace reveals particular aspects that may cause confusion and complicate decision-making. In this article, we present several less common situations underlying the general context and ECG features. The syndromes studied have a high pathological significance and may range from acute emergencies that call for a rapid therapeutical response to chronic syndromes that require prolonged observation, monitoring and risk stratification.

## 1. Introduction

It was in 1903 when the bipolar three-channel electrocardiogram (ECG) was invented and recorded by William Einthoven. Since then, ECG has been a tool of remarkable clinical power, as it can quickly provide physicians with helpful and even critical information. As ECG communicates in its own unique way, it is of paramount importance for healthcare professionals to have the necessary competency to interpret it. Thanks to some investigators having an acute eye for detail, rare cardiological syndromes have been identified and characterized by their specific ECG patterns, which may testify to their clinical presentation. The instant identification of these patterns may lead to the immediate and proper management and treatment of those patients with favorable morbidity and mortality outcomes. This review focuses on the detection of less common cardiological clinical syndromes, highlighting the general context and the ECG findings. A summary table of the syndromes’ diagnostic criteria and ECG patterns is provided below (Table 1), in order to offer a short overview of the main points and render the information more accessible to the reader.

## 2. Wellen’s Syndrome

Wellen’s syndrome was first described in 1982 by Hein J.J. Wellens (1935–2020) et al., in the Annadal Hospital, Maastricht, Netherlands, in a group of patients presenting with unstable angina. Findings later showed that 18% of the aforementioned group was proven to suffer from Wellen’s syndrome. After being solely treated medically, the majority of patients shortly developed a myocardial infarction [1].

Usually, an atheromatic plaque ruptures, travels through the bloodstream to the heart as an embolus, and temporarily occludes the left anterior descending (LAD) artery, causing a pre-infarction state [2]. This occlusion obstructs the myocardial perfusion, but soon after, the clot is lysed, and the occlusion is reversed before a myocardial infarction occurs [3].

*Clinical manifestations*. Patients commonly present with chest pain, characteristic of unstable angina, or characteristic of stable exertional angina in rarer cases. Commonly, it is a substernal pain with tightness that radiates to the left arm, the jaw, or the back. Heavy meals, cold, or exertion can exacerbate the pain [4]. The pain can also be associated with dyspnea, sweating, dizziness, and palpitations. The symptoms mainly occur in the early morning and last for about 15–25 min [5]. One atypical clinical presentation of Wellen’s syndrome was reported by Ajibawo T. et al., where a patient presented with an episode of syncope, intense abdominal pain, and vomiting and denied any of the common symptoms [6].

**Figure 1:**
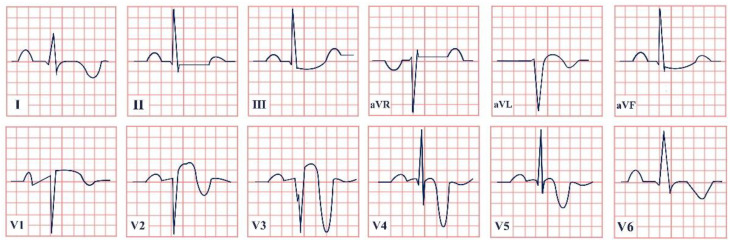
Electrocardiogram aspect in Wellen’s syndrome.

*Paraclinical diagnosis.* ECG will show a sinus rhythm and an otherwise normal ECG aspect but with T wave changes. The T wave in V2–V3 leads can be deeply inverted and symmetrical (Type A Wellen’s) or biphasic (Type B Wellen’s), as they often begin as biphasic and slowly evolve into deeply inverted ones (Figure 1). ECG can appear totally normal during the period of chest pain and only show changes when the patient is pain-free, which is why obtaining a series of ECGs is crucial to establishing the diagnosis [7].

The cardiac enzymes can be normal or slightly increased. Coronarography is positive for LAD artery occlusion in almost all cases, with few exceptions, showing a critical total or near-total obstruction. Cardiac stress testing is strictly contraindicated, as it can trigger an acute myocardial infarction [8].

*Diagnostic criteria.* Wellen’s diagnostic criteria are as follows:

1. Previous history of angina.
2. Normal or slightly increased cardiac enzymes.
3. Normal ECG during angina and T wave abnormalities when pain-free in V2–V3 (deeply inverted in Type A or biphasic in Type B).
4. No pathological Q waves.
5. No R wave loss [9].

*Differential diagnosis*. The following ECG changes can mimic the specific Wellen’s syndrome aspects.

– T wave inversion or biphasic T wave due to ischemia.
– T wave inversion, in most leads, due to Takotsubo cardiomyopathy.
– ST-segment depression and T wave flattening, best seen in V2–V4, due to hypokalemia.
– T wave inversion in V1–V6 due to acute pulmonary embolism.
– T wave inversion due to cardiac memory, such as in LBBB, pacemakers, or transient tachycardia.
– T wave inversion in V1–V3 physiologically in prepubertal patients.
– T wave inversion in most leads due to pericarditis, myocarditis, or advanced AV block [10].

*Treatment/Prognosis*. Prognosis can be poor if Wellen’s syndrome goes unrecognized and is treated with pharmacotherapy alone. Due to its similarities with acute coronary syndrome (ACS) without persistent ST-segment elevation, it can be frequently misdiagnosed and, thus, lead to fatal complications [3]. Prognosis is good in patients who undergo coronary revascularization with either percutaneous coronary intervention (PCI) to open the vessel and improve blood flow or coronary artery bypass grafting (CABG), using a vessel graft to bypass the occlusion. Patient follow-ups, identification, and elimination of atherogenic risk factors are highly suggested as there is a risk of recurrence [11].

## 3. De Winter Syndrome

De Winter syndrome is an acute coronary syndrome that is equivalent to the ST-segment elevation syndrome, caused by a left anterior descending (LAD) artery occlusion, without an apparent ST-segment elevation [12]. William Dressler et al. first described the characteristic pattern of peaked T waves and the absence of ST-segment elevation in the context of acute myocardial infarction in Maimonides Medical Center, in Brooklyn, New York, in 1947 [13]. In 2008, in the Academic Medical Center of Amsterdam, Netherlands, Robbert J. de Winter et al., further investigated the syndrome and demonstrated its presence in 2% of the patients examined [14].

The syndrome’s pathophysiology remains poorly understood to this day. A suspected mechanism postulates that the infarcted area is too substantial and can only be recorded through aVR [15]. Other suggested mechanisms include the presence of ischemia subendocardially instead of subepicardially, as well as the ATP-sensitive potassium channels in the sarcolemma not being activated, but further research remains to be carried out [16]. Lastly, it could be attributed to conduction delay due to an anatomical variability of the Purkinje fibers [15].

*Clinical manifestations.* Winter syndrome most likely occurs in males in their fifties as opposed to patients diagnosed with anterior ST-elevation myocardial infarction (STEMI) who were usually in their sixties and consistently displayed dyslipidemia [15]. The signs and symptoms are identical to typical ACS with chest tightness and pain that could radiate to the arms, shoulders, and jaw. Dyspnea, diaphoresis, vertigo, and gastrointestinal discomfort are also common clinical presentations [17,18].

**Figure 2:**
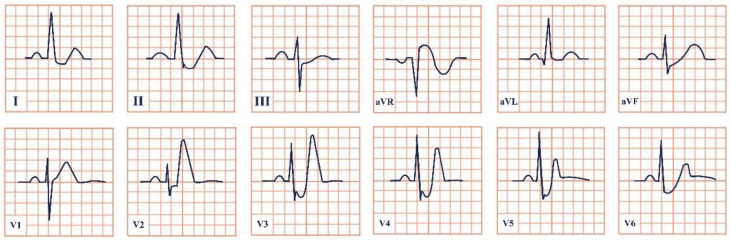
Electrocardiogram aspect in De Winter syndrome.

*Paraclinical diagnosis.* Electrocardiography is the gold standard of diagnosis, with de Winter syndrome presenting with an up-sloping ST-segment depression at the J point, tall and peaked T waves, an absence of ST-segment elevation in leads V1–V6, and a mild ST-segment elevation in lead aVR (Figure 2) [14]. At the first stages of the disease cardiac enzymes might have negative values; therefore, the serial testing of Troponin I and T, Creatinine Kinase (CK), and Creatinine Kinase-MB (CK-MB) should be conducted [19]. NT-proBNP should be evaluated daily to assess the left ventricular dysfunction and impending heart failure [Yuanyuan X., 2020] [20]. Echocardiography is an important tool aiding in the assessment of the state of the heart. According to Verouden N., echocardiography revealed mild left ventricular dysfunction in approximately 40% of the patients and severe dysfunction in approximately 16% [14]. Cardiac catheterization characteristically portrays LAD artery obstruction [18].

*Diagnostic criteria*. (1) No ST-segment elevation in precordial leads. (2) Up-sloping ST-segment depression at the J-point, which is greater than 1 mm. (3) Deep and symmetrical T-waves in the precordial leads. (4) ST-segment elevation of less than 0.5 mm in lead aVR [14]. (5) May be preceded or followed by ST-segment elevation [21].

*Differential diagnosis*. Wellen’s syndrome, acute coronary syndrome, or synthetic cannabinoids [22].

*Treatment/Prognosis*. Management should follow the acute coronary syndrome guidelines with oxygen and morphine administration in the acute setting to treat the pain, anxiety, and shortness of breath. Primary percutaneous coronary intervention or fibrinolysis can be used accordingly for LAD artery reperfusion. Anticoagulants should be administered as well [23]. The syndrome’s prognosis has improved significantly over the years, with primary PCI and fibrinolysis making it curable [16].

## 4. Kounis Syndrome

Kounis syndrome is characterized by the simultaneous occurrence of an ACS and an episode of mast cell activation, such as an allergic, anaphylactic, anaphylactoid or hypersensitivity reaction [24]. It was first described by Nikolaos G. Kounis and Georgios M. Zavras in the hospital for chest diseases in Patras, Greece, in 1991 as a “syndrome of allergic angina” [25].

A variety of causative agents has been described, ranging from environmental irritants, such as cold or exertion, to medication, for example, antibiotics, contrast media, non-steroidal anti-inflammatory drugs, muscle relaxants, or immunosuppressants [26]. The allergic reaction can be categorized as immunologic or non-immunologic, immunologic meaning an IgE-mediated reaction. Upon the initial exposure to the allergen, IgE antibodies are produced, which attach to the cardiac mast cells surface. As soon as the patient is exposed to the allergen again, the IgE antibodies start pairing up and forming bridges between them and signaling for inflammatory mediators to be released [27]. In the non-immunologic reactions, the allergens directly stimulate the mast cells, resulting in the immediate release of mediators. Kounis syndrome is divided into two main variants: type 1 is an allergic angina caused by coronary artery spasm, whereas type 2 is an allergic myocardial infarction caused by an atheromatous plaque rupture and subsequent thrombosis [26]. A third type was proposed in 2009, characterized by the thrombotic occlusion of a drug-eluting stent (DES) [28].

*Clinical manifestations*. The signs and symptoms can be partly attributed to an ACS as well as to a hypersensitivity reaction. The patients will be presented with chest pain, chest tightness, shortness of breath, and diaphoresis due to the ACS [25]. As far as the hypersensitivity reaction is concerned, the signs and symptoms can range from mucocutaneous, such as redness and urticaria, up to hypotension, tachycardia, dyspnea, gastrointestinal disturbances, arrhythmias, and bronchospasm based on the grade of severity [27].

**Figure 3:**
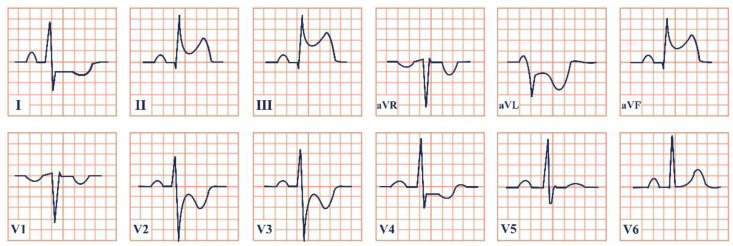
Electrocardiogram aspect in Kounis syndrome.

*Paraclinical diagnosis*. Blood samples should be collected immediately after an acute chest pain episode in the emergency room, in order to quantify histamine levels, which are only available for about 8 min. Serum tryptase is an emerging biomarker secreted by mast cells, which should be quantified every 30 min for the next 2 h, in order to rule out or confirm the allergic reaction [29]. Although increased histamine levels are characteristic, physicians might not always be able to obtain them in time and they should not be considered the gold standard for diagnosis. Serum tryptase, on the other hand, is peaking at approximately 90 min and it is a more promising biomarker [30]. Electrocardiographic findings can be nonspecific, with the most common resembling the ECG changes of digoxin toxicity, such as ST-segment depression, atrial fibrillation, bigeminal rhythm, QRS complex prolongation, QT-segment prolongation, ventricular ectopic beats, T wave inversion or flattening, sinus tachycardia, AV block, sinoatrial block, or ventricular fibrillation (Figure 3) [31]. The cardiac enzymes (troponins, CK, CK-MB) will be elevated, due to the myocardial injury. Echocardiography and coronary angiography should be performed to rule out any existing or newly installed pathologies of the myocardium and to visualize the coronary arteries. Single-photon emission computed tomography (SPECT) using thallium-201 and 125I-15-(p-iodophenyl)-3-(R, S) methylpentadecanoic acid can also be used, which will reveal the myocardial ischemia. Dynamic MRI and delayed contrast-enhanced MRI can contribute to the diagnosis [29].

*Diagnostic criteria*. Kounis syndrome should be suspected in young and healthy patients who are not presumed to have atherosclerosis and who are presenting with ACS [32]. In order to establish a positive diagnosis, the patient must present with a systemic allergic reaction accompanied by myocardial ischemia demonstrated by the characteristic electrocardiographic, laboratory, ultrasound, and angiographic findings [29].

*Differential diagnosis*. Acute coronary syndrome, scombroid poisoning, or carbon monoxide intoxication [33,34].

*Treatment/Prognosis*. Prognosis can differ according to the type of Kounis syndrome; type 1 can be more favorable than type 2. Nevertheless, the severity of the initial allergic reaction, the patient’s susceptibility and comorbidities, and the allergen concentration all influence the prognosis [24]. In the first type, treating the allergic reaction with antihistaminics and corticosteroids is enough. In type 2, not only the allergic reaction needs to be treated but also the underlying myocardial infarction. Caution must be given to beta-blockers, which can worsen the vasospasm, as well as to adrenaline, which would otherwise be the gold standard of treatment but exacerbates the ischemia and can intensify the vasospasm in Kounis. In type 3, all previous measures must be taken, as well as the urgent aspiration of the stent thrombus [31].

## 5. Takotsubo (Broken Heart) Syndrome

Takotsubo syndrome, also named stressed-induced cardiomyopathy, “broken heart” syndrome or apical ballooning, with the most common term being Takotsubo, due to the resemblance of the heart’s left ventricle to a Japanese fisherman’s pot for trapping octopuses [35]. Patients present with a paroxysmal malfunction of the left ventricle, characterized by the apical distention and immobility and basal hyperkinesia of the left ventricle [36], which is not the result of an acute myocardial infarction but sometimes the two may coexist [37]. It is a reversible syndrome, with cardiac function return in approximately 12 weeks and the normalization of electrocardiogram and biomarker levels in 6–12 months [38]. The syndrome was first described by Sato et al. in 1990 in Hiroshima, Japan [39] and further explained in 1991 by Dote et al. [40].

A variety of pathophysiological mechanisms have been described over the years. In many cases, multivessel coronary vasospasm with an associated catecholamine surge has been reported. In other cases, only a 10–20 times higher serum catecholamine level has been observed, either due to a catecholamine injection or due to an underlying pathology, such as pheochromocytoma, thyrotoxicosis and sympathetic storming after traumatic brain injury. Hypotheses suggest that due to the larger number of beta-adrenergic receptors in the apex, as opposed to the base of the heart, the apex is more sensitive to catecholamines and can therefore become stunned. Another hypothesis suggests that, since estrogen downregulates the beta-adrenoreceptors’ expression, post-menopausal women will present with an accentuated response to serum catecholamines [41].

*Clinical manifestations*. The syndrome can be triggered by emotional as well as physical stimuli or can appear without any obvious stimulus at all. It is associated with heartbreaking events, for example, the death of a loved one, an economic crisis, or a war, but it can very well appear in moments when the patient is overjoyed, such as athletic tournaments [37]. The prevalence of a psychiatric or neurological disorder is significantly higher in Takotsubo patients. As far as physical stimuli are concerned, the cause could be an acute disease or a medical procedure or treatment imposing the patient under stress, such as surgery, cardiac stress testing, or chemotherapy [41]. The symptoms are that of an ACS with dyspnea, chest pain at rest radiating to the jaw, diaphoresis, nausea, dizziness, and the symptoms of the underlying disease if there is one [42].

**Figure 4:**
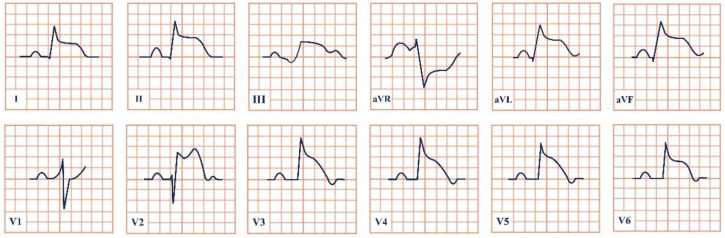
Electrocardiogram aspect in Takotsubo syndrome.

*Paraclinical diagnosis.* Patients in the acute onset of the disease will typically present with ST-segment elevation (Figure 4), which will require further cardiac catheterization with coronary angiography, left ventriculography, and invasive left ventricle volume and pressure measurement in order to distinguish it from acute myocardial infarction or aborted myocardial infarction [43]. In the majority of Takotsubo patients, there is no angiographic evidence of coronary obstruction, although there is a small percentage of cases where the two disorders may coexist [36]. In the upcoming 24–48 h, patients can present with deep T wave inversion, QT-interval prolongation, and pathological Q waves [41]. Continuous ECG monitoring is suggested, as progressive QT-interval prolongation can lead to lethal arrhythmias, including Torsades de Points and ventricular fibrillation. In cases without ST-segment elevation, the physician should take into consideration the International Takotsubo (InterTAK) diagnostic criteria and consider the probability of Takotsubo [43].

The cardiac enzymes are elevated but on the lower side in comparison to an acute myocardial infarction. The left ventricular dilation will mediate the transient increase of BNP and NT-proBNP with a peak at 24–48 h [43]. Serum catecholamines can also be measured and they usually present with a two-fold increase. Imaging methods can be used, such as echocardiography, cardiac computer tomography angiography, cardiac magnetic resonance, and myocardial perfusion scintigraphy, to evaluate the left ventricular function and to rule out any potential complications [41].

*Diagnostic criteria.* The InterTAK score takes into consideration seven parameters that can be easily evaluated in the Emergency Room for risk stratification. Each criterion is allocated several points and if their sum is greater or equal to 70, the patient is considered to have a high risk of suffering from Takotsubo and vice versa. Female sex is given 25 points; the presence of an emotional stressor is given 24; physical stressor, 13 points; the absence of an ST-segment depression is allocated 12 points (except for aVR); the presence of a psychiatric disorder is given 11 points; a neurological disorder, 9 points; and QT-interval prolongation is assigned 6 points [43].

The Heart Failure Association of the European Society of Cardiology has proposed another set of criteria, where all seven parameters must be met: (1) Transient kinetic wall alterations, which may be brought on by an emotional or physical stressor. (2) New and reversible ECG changes (ST-segment elevation, ST-segment depression, left bundle branch block, T-wave inversion, and/or QTc prolongation) during the acute phase (3 months). (3) The absence of evidence of causative atherosclerotic coronary artery disease, including acute plaque rupture, thrombus formation, and coronary dissection or other pathological conditions to explain the temporary LV dysfunction (e.g., hypertrophic cardiomyopathy, viral myocarditis). (4) The kinetic wall alterations commonly extend beyond a single coronary vessel territory and cause the circumferential dysfunction of the cardiac segments involved. (5) Important increase in serum natriuretic peptide (BNP or NT-proBNP) during the acute phase. (6) Small raise in cardiac troponin measured with a conventional assay. (7) The rehabilitation of ventricular systolic function on cardiac imaging at follow-up (3–6 months) [38].

*Differential diagnosis*. Acute coronary syndrome, myocardial infarction with non-obstructive coronary arteries (MINOCA), or Kounis syndrome.

*Treatment/Prognosis*. Upon presentation, the differentiation with ACS cannot be made and the patient will be treated with aspirin, clopidogrel, intravenous heparin, fibrinolytics, morphine, and oxygen. Upon confirmation of the syndrome, patients will be treated according to severity. Mild cases can be treated with angiotensin-converting enzyme (ACE) inhibitors or angiotensin receptor blockers (ARBs), to potentially reverse the dilation and beta-blockers to antagonize the catecholamine activity. In heart failure and pulmonary edema, diuretics and nitroglycerin should be added in the absence of left ventricular outflow tract obstruction (LVOTO). Severe cases of cardiogenic shock should be transferred to the intensive care unit (ICU) and possibly treated with a left ventricular assist device. Any underlying conditions and complications should be sought out and treated accordingly [43]. Although prognosis is thought to be good with most patients making a complete recovery, the syndrome is unfortunately associated with increased complications and the high risk of mortality and a recurrence rate of 22% [44].

## 6. Yamaguchi Syndrome

Yamaguchi syndrome, or Left Ventricular Apical Hypertrophy, is a hypertrophic non-obstructive cardiomyopathy with a predilection for the left ventricular apex. It was first described by Tsuguya Sakamoto et al. in 1976 in Tokyo, Japan [45]. In 1979, Hiroshi Yamaguchi et al. further explained the syndrome by adding ventriculographic evidence and a larger sample of patients as well, at the Toranomon Hospital, in Tokyo, Japan [46].

Yamaguchi syndrome is a hereditary disease with an autosomal dominant transmission. The defect is found in the genes encoding the sarcomere or sarcomere-related proteins, compromising the physiological inotropy of the heart and, therefore, causing hypertrophy [47]. The hypertrophy mainly involves the apex of the left ventricle in the pure form of the disease, in case other segments of the heart are involved, the hypertrophy is classified as “mixed” [48]. Yamaguchi tends to occur more often in males and patients older than 40 years of age [49].

*Clinical manifestations.* Patients exhibit symptoms that are similar to acute coronary syndrome (ACS), such as angina and dyspnea. They can also exhibit chest pain that worsens with movement and is relieved by rest, syncope, and palpitations [49]. According to Eriksson et al., in many patients, the presenting symptoms were those of the complications of the disease. In a sample of fifty-seven patients, ten of them were first presented with atrial fibrillation, five with ACS with ST-segment elevation, and the last two with exercise-induced ventricular fibrillation and heart failure accordingly. The rest of the patients did not have any specific symptoms and their diagnosis was secondary to a medical check-up [48].

**Figure 5:**
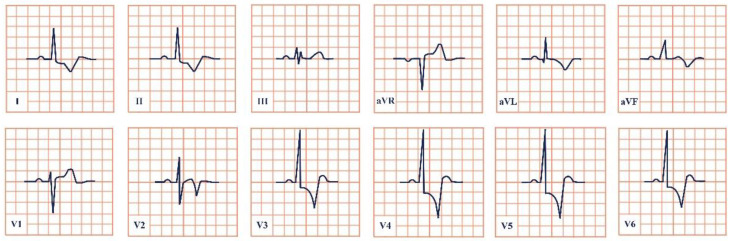
Electrocardiogram aspect in Yamaguchi syndrome.

*Paraclinical diagnosis*. Electrocardiography will reveal characteristically giant negative T waves, greater than 10 mm, and left ventricular hypertrophy, which can be confirmed using the Sokolov–Lyon criteria and calculating whether the sum of the S wave in V1 and the R wave in leads V5, V6 is greater than 35 mm [50]. These ECG changes should be presented as stand-alone and without the presence of an important underlying coronary artery disease or hypertension. ECG may also reveal signs of complications, including atrial fibrillation and ACS with ST-segment elevation (Figure 5) [47].

As far as blood testing is concerned, troponins I and T may be elevated [49]. Cardiac scintigraphy along with a stress test will reveal reversible ischemia of the heart, accentuated with exertion. Yamaguchi exhibits a typical pattern on ventriculography, with the left ventricle resembling an ace of spades [51]. Echocardiography must be performed to quantify all the segments of the heart and confirm the apical hypertrophy, display whether the hypertrophy is “pure” or “mixed” and possible complications. Gadolinium Enhanced Cardiac Magnetic Resonance (CMR) can be used in case the diagnosis is inconclusive [48]. Coronary angiography is crucial in order to exclude acute coronary syndrome with ST-segment elevation, as the two may coexist [46].

*Diagnostic criteria*. The diagnosis is established with the following imagistic criteria:

1. Presence of asymmetric left ventricular hypertrophy.
2. Left apex thicker than 15 mm.
3. Ratio of apical wall thickness to posterior wall thickness higher than 1,5 [48].

*Treatment/Prognosis.* Patients suffering from Yamaguchi complicated with heart failure could benefit from a biventricular pacemaker to improve the ventricular dyssynchrony and increase the left ventricular diastolic filling [52]. Yamaguchi has quite a high morbidity of approximately 16% and a mortality of 10%. According to Eriksson et al., only 44% of patients examined periodically were asymptomatic after a period of 5 years. The rest of the patients had various degrees of heart failure symptomatology, with 20% of them gradually deteriorating [48].

## 7. Wolff–Parkinson–White Syndrome

Wolff–Parkinson–White (WPW) syndrome is a ventricular pre-excitation syndrome due to an accessory conduction pathway. Patients can exhibit the syndrome, meaning that they suffer from symptoms and display the typical ECG changes. Patients who have the Wolff–Parkinson–White pattern only display ECG changes without reporting any symptoms [53]. WPW syndrome was first reported as a syndrome with a shortened PR interval by Louis Wolff, John Parkinson, and Paul White in 1930, physicians in Boston, Massachusetts, and London, the United Kingdom, in a case study of eleven athletic and otherwise healthy patients [54]. Shortly after, in 1932, Holzmann and Scherf attributed the syndrome to a bypass tract [55].

Accessory pathways can differ from individual to individual, anatomically and electrophysiologically. Anatomically, they can be atrioventricular, such as the bundle of Kent, internodal between the sinoatrial and atrioventricular node, atriofascicular, nodoventricular, and nodofascicular. As far as electrophysiology is concerned, these pathways create a bypass route for impulse conduction, which can be antegrade, retrograde or bidirectional, also called manifest pathways [56]. The most common accessory pathway in Wolff–Parkinson–White is the bundle of Kent [57]. Wolff–Parkinson–White has been associated with congenital heart defects, with the most common being Ebstein’s anomaly and has been shown to have an autosomal dominant transmission, although that is not always the case [58].

*Clinical manifestations.* Patients can complain of palpitations, syncope, dizziness, chest pain, dyspnea, unexplained anxiety, or can sometimes be asymptomatic [59]. Males are more commonly affected. Symptoms may be triggered by emotions, exertion, or pregnancy in women [60]. Atrial fibrillation and flutter, paroxysmal supraventricular tachycardia, ventricular fibrillation, and cardiac arrest can appear secondary to the syndrome [61].

**Figure 6:**
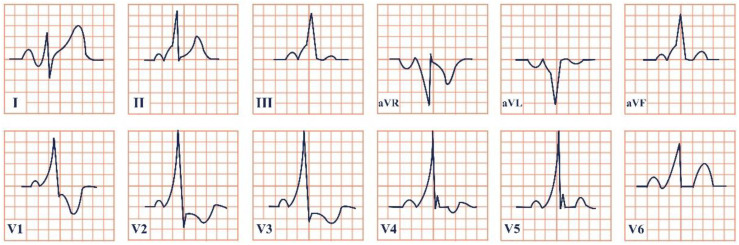
Electrocardiogram aspect in Wolff–Parkinson–White syndrome.

*Paraclinical diagnosis*. On ECG, in sinus rhythm, patients can either display the typical pattern of a shortened PR interval, delta wave, and widened QRS complex or have no indication of presence of the syndrome if the pathway is strictly retrograde or not excited during the evaluation (Figure 6) [58,62]. Secondary arrhythmias may also be present [61]. Holter monitoring and serial ECGs are recommended along with cardiac stress testing, especially in patients with the WPW pattern for risk stratification [63]. Intracardial electrophysiological testing (EPT) is recommended for symptomatic patients and asymptomatic patients with a high-risk line of work, including athletes, pilots, and drivers, to localize the pathway, its direction, and ablate it [57].

*Diagnostic criteria.* Diagnosis is established with the presence of the following ECG changes: (1) Short PR interval less than 120 ms. (2) Delta wave, meaning a slurred upstroke at the beginning of the QRS complex. (3) Wide QRS complex greater than 120 ms. (4) T wave and ST-segment changes secondary to the syndrome [64]. The QRS complex can take different forms depending on the location of the accessory pathway, diving WPW into two types. Type A is characterized by a positive delta wave in V1; positive QRS complex in leads V1, II, III, and aVF; and negative or isoelectric Q waves in the lateral leads. Type B is characterized by negative delta waves and positive upright QRS complexes in leads II, III, and aVF [65].

*Differential diagnosis.* Lown–Ganong–Levine syndrome, acute coronary syndrome, or ventricular hypertrophy.

*Treatment/Prognosis.* Acute cases should be treated according to the tachyarrhythmia present. Long-term, physicians should determine the patient’s risk stratification. Asymptomatic patients who are not in risky occupations are observed, while the rest of the patients should undergo interventional catheter ablation. Pharmacotherapy with a combination of beta-blockers and non-dihydropyridinic calcium channel blockers or beta-blockers and antiarrhythmics is given, in case ablation is contraindicated [53]. Wolff–Parkinson–White is a well-recognized syndrome which can be asymptomatic. Symptomatic individuals have many options for treatment, making the overall prognosis excellent [57].

## 8. Lown–Ganong–Levine Syndrome

Lown–Ganong–Levine syndrome or Enhanced AV Nodal Conduction is a ventricular pre-excitation syndrome with an uncertain underlying etiology. Clerc, Levy, and Critesco were the first to report the syndrome in 1938 but thought it a variant of Wolff–Parkinson–White [66]. In 1952, Bernard Lown, William F. Ganong, and Samuel A. Levine working in the Peter Bent Brigham Hospital, in Boston, Massachusetts, further described the syndrome as a distinct entity [67].

The syndrome’s etiology is ambiguous. It has been attributed to the presence of accessory pathways, as well as to congenital atrioventricular node hypoplasia [68]. Although the exact mechanism is not fully known, the two anatomical variants of the bypass tracts are suspected: the presence of the James or Paladino fiber, situated between the sinoatrial and atrioventricular nodes, and the presence of the Brechenmacher fiber, a bypass tract located between the sinoatrial node and His bundle [69]. Current research has not been able to pinpoint an accessory pathway common to all those affected [70].

*Clinical manifestations*. Patients are usually females older than 30 years of age. They complain of tachycardia, chest pain, syncope, and palpitations [67,68]. In some cases, atrial fibrillation, atrial flutter, ventricular fibrillation, or cardiac arrest and sudden death can occur [70].

**Figure 7:**
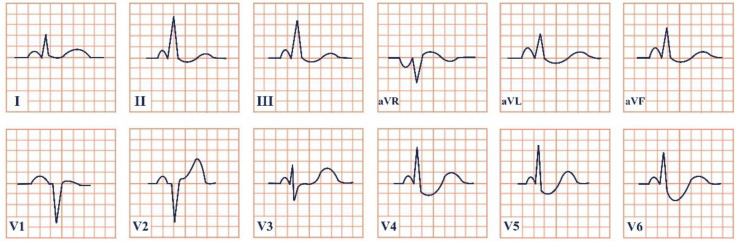
Electrocardiogram aspect in Lown–Ganong–Levine syndrome.

*Paraclinical diagnosis.* In electrocardiography in sinus rhythm, the syndrome is characterized by supraventricular tachycardias, a short PR interval, and a normal QRS complex without a slurred upstroke (Figure 7). Secondary arrhythmias may also be seen [71]. Electrophysiologic studies with premature atrial stimulation will provide data about the refractory period and may reveal the underlying accessory pathway [72].

*Diagnostic criteria*. The following electrocardiographic criteria have been proposed, although they are not present in all patients and cannot always be considered reliable: (1) Short PR interval, less than 120 ms. (2) Normal QRS complex, smaller than 120 ms. (3) Absence of delta wave [67,73]. This set of electrophysiologic criteria has also been suggested, although they may also be arbitrary: (1) The A-H interval (defined as the conduction time from the atria to His bundle) in sinus rhythm is smaller or equal to 60 ms. (2) 1:1 A-V conduction is maintained during atrial pacing at cycle lengths less than 300 ms (200/min). (3) The maximum A-H interval prolongation during atrial pacing is smaller or equal to 100 ms [72].

*Differential diagnosis.* Wolff–Parkinson–White syndrome.

*Treatment/Prognosis.* Patients are commonly treated with beta-blockers and antiarrhythmic drugs. Catheter ablation can be used in selected cases, where the accessory pathway is identified [74]. The syndrome can lead to ventricular fibrillation and sudden death at rest, but this is a rare finding among patients, making the overall prognosis of Lown–Ganong–Levine syndrome quite good [70].

## 9. Brugada Syndrome

Brugada syndrome is an autosomal dominant syndrome, associated with sudden cardiac death in families, without the presence of any underlying gross structural heart disease [75]. This syndrome was first reported in Thailand in 1917 by Guazon M. as “bangungut” meaning the unexplained sudden death of young male patients with structurally normal hearts upon autopsy [76]. In 1992, Pedro and Josep Brugada, in the Cardiovascular Center of OLV Hospital in Aalst, Belgium, and the Cardiology Department of the Hospital Clinic in the University of Barcelona, Spain, accordingly, further investigated the syndrome and presented it as a separate entity [77].

There has been a vast variety of mutations associated with Brugada, the first one discovered being SCN5A, found in chromosome 3p21-24. It is present in approximately 15–30% of Brugada patients [78]. Over the years, there have been three pathophysiological mechanisms proposed. The repolarization hypothesis suggests that there is a functional sodium channel mutation that results in a decreased sodium current and a gradient between the epicardial and endocardial action potential. As the gradient increases, cells may begin repolarizing near cells that are still depolarized, therefore causing a re-entrant arrhythmia [75]. The depolarization hypothesis suggests that the right ventricular outflow tract (RVOT) undergoes certain microscopic changes, such as fibrosis, myocarditis, or apoptosis, which causes a delayed conduction through the RVOT and a delayed depolarization. Finally, the neural crest hypothesis argues that the two previous mechanisms can coexist, since the RVOT may have a different embryological origin from the rest of the heart and possess the characteristics mentioned above [75,79].

*Clinical manifestations.* Affected individuals are usually Asian, male, and around 40 years of age with a family history of sudden cardiac death. They are usually asymptomatic [80]. Characteristic manifestations are syncope, seizures, arrhythmias, agonal breathing between midnight and early morning, and sudden cardiac death [81]. Fever, large meals, exertion, potassium abnormalities, hypercalcemia, alcohol or cocaine intoxication, and certain drugs can unmask the syndrome and produce symptoms [82].

**Figure 8:**
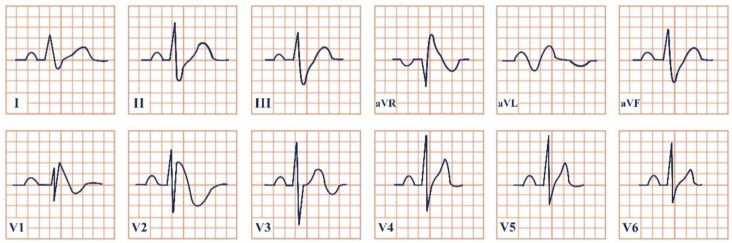
Electrocardiogram aspect in Brugada syndrome type 1.

*Paraclinical diagnosis.* Electrocardiography may reveal three characteristic patterns. Type 1 consists of coved ST-segment elevations greater than 2 mm followed by an inverted T wave (Figure 8), type 2 consists of a saddle-back ST-segment elevation greater than 2 mm followed by a normal or biphasic T wave, and type 3 is characterized by a coved or saddleback-shaped ST-segment elevation that is equal or less than 1 mm. Only type 1 is considered diagnostic, type 2 and 3 can raise the physician’s suspicion and lead to further testing. A provocation test using mainly flecainide or ajmaline or a treadmill test may be used to reveal the ECG pattern [80,83].

*Diagnostic criteria.* The presence of at least one of the following criteria confirms the diagnosis: (1) Positive family history—family member presenting Brugada pattern type 1 or deceased due to sudden cardiac death before the age of 45. (2) Symptoms related to arrhythmias: syncope, nocturnal agonal respiration, or seizures. (3) Ventricular arrhythmias: ventricular fibrillation or polymorphic ventricular tachycardia [80].

*Treatment/Prognosis.* The first-line management of Brugada syndrome is the implantation of a cardioverter-defibrillator (ICD) to prevent the occurrence of sudden cardiac death [80]. Phosphodiesterase-3 inhibitors and quinidine may be used in case of patient refusal or contraindication to ICD. An emerging and quite promising treatment is the radiofrequency ablation of the RVOT, which has been shown to normalize the ECG findings and prevent fatal ventricular arrhythmias [80,84].

## 10. Frederick’s Syndrome

Frederick’s syndrome is a disorder characterized by atrial fibrillation or atrial flutter and complete heart block occurring simultaneously [85]. It is a syndrome with a rare occurrence, first described by Leon Fredericq in the Institute of Physiology at the University of Liege, Belgium, in 1904 [86].

Leon Fredericq experimented using animal models and confirmed that by damaging the His bundle and mechanically stimulating the ventricles, the ventricles will contract at a normal rate and regular rhythm, while the atria will go into fibrillation. Thus, he confirmed the hypothesis that the syndrome can be secondary to a disease impairing the impulse conduction from the atria to the ventricles [87]. According to Trekina N., Frederick’s is usually caused due to a structural heart disease, such as cardiomyopathy, myocarditis, or acute coronary syndrome (ACS) with ST-segment elevation [88].

*Clinical manifestations.* Patients have been usually previously diagnosed with atrial fibrillation. While their heart rate is still within 60 beats per min, they will only exhibit symptoms of fibrillation, including palpitations, chest pain, fatigue, and dyspnea. Once their heart rate falls to 20–30 beats per min, the complete heart block has appeared, and their general state diminishes. Dizziness, syncope, asystole, and death can occur [88].

**Figure 9:**
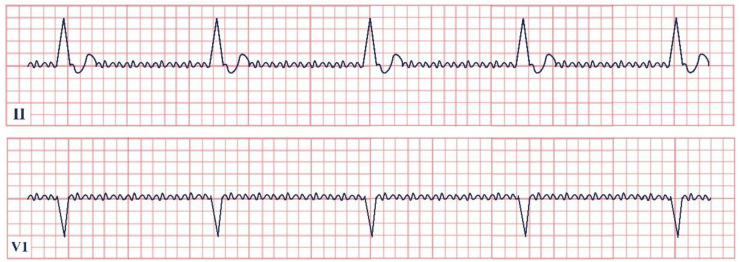
Electrocardiogram aspect in Frederick’s syndrome.

Paraclinical diagnosis. On electrocardiogram, Frederick’s will reveal atrial fibrillation or atrial flatter and a complete heart block (Figure 9). On echocardiography, biatrial enlargement is shown, as is the hypertrophy of the interventricular septum and the decreased ejection fraction [88]. Patients should be further evaluated with Cardiac Magnetic Resonance (CMR) for the underlying cause of the syndrome [89].

*Diagnostic criteria.* Electrocardiography is the gold standard for diagnosis. The following criteria may be used: (1) Absent P waves. (2) Presence of atrial fibrillation f waves or atrial flutter waves. (3) Third degree atrioventricular block characterized by AV dissociation [85].

*Prognosis.* Frederick’s is an underdiagnosed and underreported syndrome in the English-language literature. Its prognosis is not fully known due to the lack of data. However, since it has a high morbidity and its underlying cause is not often found, its prognosis can be poor [88].

## 11. PAFIYAMA Syndrome

Paroxysmal atrial fibrillation in young and middle-aged adults (PAFIYAMA), is a syndrome characterized by the presence of recurrent self-limited episodes of atrial fibrillation (AFib) in young and middle-aged athletes who have been practicing strenuously a particular sport for a prolonged amount of time. The term PAFIYAMA was first introduced by Fabian Sanchis-Gomar et al. in 2017 at the University of Valencia, Spain [90].

Strenuous long-term training leads to the dilation of all four cardiac cavities, also called athlete’s heart or Phidippides cardiomyopathy after the Greek runner who died after running 240 km to announce the victory of war [91]. The ventricular walls are thicker and much more resistant to stress than the atrial ones, leading to atria remodeling, inflammation, and eventually fibrosis. Athletes have more pronounced parasympathetic activity, which shortens the trial refractory time, therefore facilitating higher atrial firing rates. Another suggested mechanism is the remodeling of the sinoatrial node in order to decrease the resting heart rate. Pulmonary vein ectopy has also been shown to be a trigger for AFib [90].

*Clinical manifestations.* Atrial fibrillation can be commonly asymptomatic (silent AFib). Patients may develop palpitations, dyspnea, exercise intolerance, dizziness, fatigue, presyncope, or syncope [92,93].

**Figure 10:**
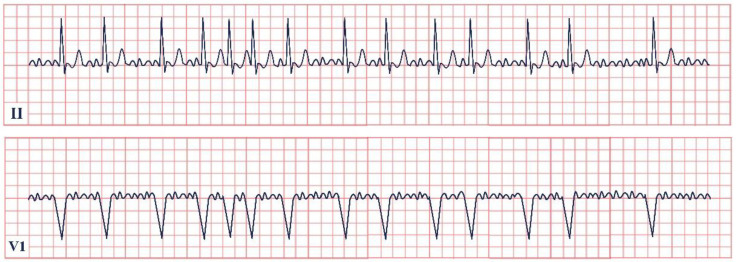
Electrocardiogram aspect in PAFIYAMA syndrome.

*Paraclinical diagnosis.* Serial ECG testing and 7-day Holter ECG monitoring are crucial for the detection of AFib and the exclusion of other cardiac syndromes (Figure 10). Cardiac stress testing is recommended in order to trigger the atrial fibrillation in suspected cases which are not yet confirmed [91]. Blood tests to exclude hyperthyroidism, diabetes mellitus, and pheochromocytoma should be conducted. Echocardiography is crucial to visualize any structural heart defects [90].

*Diagnostic criteria.* The following criteria are recommended for diagnosis. To confirm the diagnosis, all the major and at least five of the minor criteria should be fulfilled.

Major criteria: (1) Preserved ejection fraction (≥55%). (2) Patient is less than 60 years old and of male sex. (3) Long-term strenuous training (more than 6–8 h weekly performing with 60% of maximum heart rate, for more than 6 months). (4) Onset as paroxysmal AFib.

Minor criteria: (1) Normal, or even supranormal, diastolic function. (2) Inverted T waves in 2 leads. (3) Enhanced parasympathetic system (prolonged PQ time, sinus bradycardia, first degree AV-block). (4) Left atrial enlargement. (5) Left ventricular hypertrophy. (6) Thickened left ventricle, increased in size. (7) ST-segment elevation at the J-point greater than 0.1 mm in 2 leads.

The exclusion of usual risk factors for atrial fibrillation: (1) Body weight index less or equal to 25 kg/m^2^. (2) Normotensive at rest. (3) No smoking. (4) No diabetes mellitus.

The exclusion of underlying diseases: (1) Electrolyte disturbances. (2) Dilated or hypertrophic cardiomyopathy. (3) Obstructive sleep apnea. (4) Coronary artery disease. (5) Wolff–Parkinson–White syndrome, Brugada syndrome, long QT syndrome, arrhythmogenic cardiomyopathy, or catecholaminergic ventricular tachycardia. (6) Performance-enhancing agents or illicit drug use. (7) Pericarditis. (8) Metabolic or hormonal diseases (hyperthyroidism, pheochromocytoma) [90].

*Treatment/Prognosis.* The reduction of strenuous training in volume and intensity is highly recommended and has been shown to reverse the myocardial hypertrophy. Athletes are advised to stop training for at least two months. Pharmacotherapy is the first-line approach. Rhythm control may be used alongside anticoagulant prophylaxis, and rate control is avoided as it could decrease the athlete’s performance. Direct-current cardioversion and circumferential catheter ablation of the pulmonary vein are the last line and may be used to restore the sinus rhythm [94]. The cessation or reduction of exercise may seem debilitating to professional athletes, and it is possible that they will refuse to do so. Personalized treatment and psychological counseling are, therefore, highly recommended. These patients have a very low CHA_2_-DS_2_-VASc score, as well as a lower mortality risk in comparison to other atrial fibrillation patients, rendering their overall prognosis good [95].

## 12. Haïssaguerre Syndrome

Haïssaguerre syndrome, also named J wave syndrome or early repolarization syndrome, is characterized by the presence of early repolarization, idiopathic ventricular fibrillation and sudden cardiac death [96]. J wave abnormalities were first reported by Tomaszewski, W., in 1938 in a hypothermic patient [97]. There had been many reports of early repolarization in the literature, but the syndrome was thought to be benign until 2008. In 2008, Haïssaguerre, Michel, et al., from the university hospital Hôpital Haut-Lévêque, Bordeaux-Pessac, in Bordeaux, France, and Rosso, Raphael, et al. from the Tel Aviv and Sheba Medical Center in Tel Aviv, Israel, both demonstrated the syndrome’s correlation with arrhythmogenicity [98,99].

As the name suggests, early repolarization syndrome can be attributed to a genetic loss or gain of function channel mutation leading to a transmural gradient between epicardium and endocardium and an earlier repolarization, similarly to Brugada and Osborn syndromes. Some claim that the syndrome can be caused by late depolarization due to delayed activation and conduction [100]. The early repolarization hypothesis is more strongly supported, considering that the J wave decreases with tachycardia [101].

*Clinical manifestations.* The syndrome affects young males more commonly but starting from adulthood its male predominance decreases [102]. Patients are usually athletes or individuals with an active lifestyle and of African American origin [103]. They present with unexplained syncope, polymorphic ventricular tachycardia, and idiopathic ventricular fibrillation that may have led to resuscitated cardiac arrest or have a family history of sudden cardiac death [100,104].

**Figure 11:**
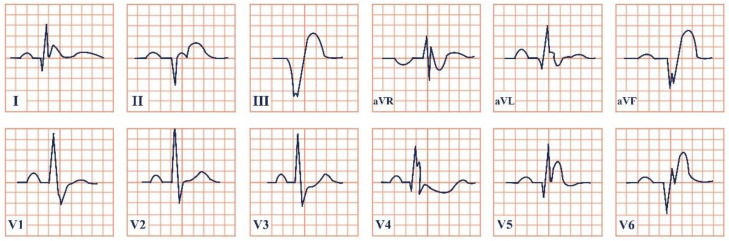
Electrocardiogram aspect in Haïssaguerre syndrome.

*Paraclinical diagnosis.* According to Andelevitch, C., there are three distinct types of the syndrome (Figure 11). Type 1 is commonly seen in young athletes in leads I, V4–V6 and has a more benign course; type 2 is seen in leads II, III, and aVF and has been associated with a moderate risk; and type 3 is seen in all leads and is the one highly associated with malignant arrhythmias [105]. A provocation test using ajmaline or a cardiac stress treadmill test may be utilized to show any malignant ECG patterns, as the benign forms usually disappear during these tests [106]. A 24 h Holter ECG is suggested in unexplained ventricular fibrillation to identify the characteristic pattern, as well as echocardiography, cardiac MRI, coronary angiography, and biopsy to exclude any other causes [103].

*Diagnostic criteria.* The following diagnostic criteria were established by the 2013 Expert Consensus Statement by the Heart Rhythm Society (HRS), the European Heart Rhythm Association (EHRA), and the Asia Pacific Heart Rhythm Society (APHRS):

1. Haïssaguerre syndrome diagnosis is confirmed by a J-point elevation larger than 1 mm in two or more contiguous inferior and/or lateral leads in a patient with a history of resuscitation from otherwise unexplained ventricular fibrillation or polymorphic ventricular tachycardia.
2. Haïssaguerre syndrome can be diagnosed in a deceased patient due to sudden cardiac death who has no cardiac structural defects upon autopsy and a previous ECG with a J-point elevation larger than 1 mm in two or more contiguous inferior and/or lateral leads.
3. Haïssaguerre syndrome can be diagnosed with the presence of a J-point elevation larger than 1 mm in two or more contiguous inferior and/or lateral leads on ECG [103].

*Treatment/Prognosis.* Patients with an early repolarization pattern present only in the precordial leads are considered very low risk for malignant arrhythmias and have an excellent prognosis. On the other hand, patients with the pattern present in the inferior and/or lateral leads have a higher risk. The magnitude of the wave plays an important role as well: the larger the wave, the higher the risk of arrhythmogenicity [101]. The implantation of a cardioverter defibrillator is recommended in symptomatic patients and patients with aborted sudden cardiac death. Isoproterenol and quinidine may be added to prevent episodes of ventricular fibrillation [103].

## 13. Osborn Syndrome

Osborn waves have been referred to by a variety of names in the literature: K, J, or H waves; camel hump sign; late delta wave; and hat-hook junction. In 1938, Tomaszewski, W., first reported the characteristic electrocardiographic changes in a hypothermic patient. In 1953, John J. Osborn from the Department of Pediatrics at the New York University College of Medicine extensively described and attributed the pattern to hypothermia, having carried out research in vivo [97,107,108,109].

Similarly to Brugada and Haïssaguerre syndromes, the two hypotheses of early repolarization and late depolarization are suspected as the causative mechanism. According to the repolarization hypothesis, there is a transmural gradient between the epicardium and endocardium which can be triggered by hypothermia, ischemia, hypercalcemia, diabetic ketoacidosis, hypothyroidism, sepsis, brain death, and drowning [108,109]. Others believe that the syndrome may be induced by a late depolarization caused by delayed activation and conduction, although this argument is not highly supported [108].

*Clinical manifestations*. Osborn waves can be present in a variety of processes, therefore rendering careful and thorough history taking of great importance. They are very commonly found in patients undergoing therapeutic hypothermia, as well as accidental hypothermia. Diabetic ketoacidosis, hypothyroidism, sepsis, brain death, Parkinson’s disease, clozapine and baclofen overdose, and drowning can also be found in patients with Osborn waves. Patients may be presented with acute coronary syndrome, hypercalcemia, Takotsubo cardiomyopathy, aborted cardiac arrest, myocarditis, concentric hypertrophy, Brugada, and Haïssaguerre, causing the characteristic ECG pattern. Since Osborn waves are not pathognomonic by themselves, patients should therefore be further evaluated [109].

**Figure 12:**
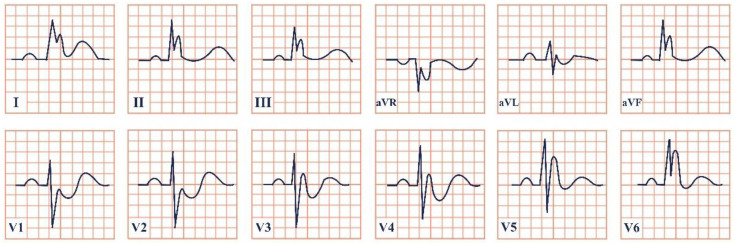
Electrocardiogram aspect in Osborn syndrome.

*Paraclinical diagnosis.* The J point on electrocardiogram is located in between the QRS complex and the ST-segment and marks the end of depolarization and beginning of repolarization (Figure 12). When the J point is no longer isoelectric and becomes positive, it is referred to as the J wave [110]. As Osborn waves can be present in several pathologies, utilizing ECG to differentiate between hypothermic and non-hypothermic causes is crucial. Hypothermic patients exhibit bradycardia, longer PR interval, QT-interval, and QRS complex and may have shivering artifacts if the hypothermia is not due to brain trauma [109]. The greater the hypothermia, the steeper the Osborn waves. Another important differentiation is that of Brugada and Haïssaguerre, where their diagnostic criteria will be evaluated [111].

*Diagnostic criteria.* The diagnostic criteria for Brugada and Haïssaguerre syndromes can be used [111].

*Prognosis.* The prognostic value of Osborn waves in hypothermic patients is debatable, some authors claim that their presence is associated with higher mortality and others that hypothermic patients who did not exhibit Osborn waves had a worse prognosis. The presence of Osborn waves due to non-hypothermic causes is associated with poorer prognosis, depending on the ECG leads associated and the magnitude of the waves [111].

## 14. LEOPARD Syndrome

LEOPARD (Lentigines, ECG abnormalities, Ocular hypertelorism, Pulmonary stenosis, Abnormal genitalia, Retardation of growth, Deafness) syndrome, also named Noonan syndrome with Multiple Lentigines, Gorlin syndrome, Multiple Lentigines syndrome, Cardio-cutaneous syndrome, Moynahan syndrome, Lentiginosis profusa, or Progressive Cardiomyopathic Lentiginosis, is a rare autosomal dominant syndrome with multiple congenital abnormalities [112]. The syndrome was first interpreted by Zeisler, E.P., et al. in 1936 [113]. It was further described and given the name “LEOPARD” in 1969 by Robert J. Gorlin, Ray C. Anderson, and Michael Blaw, working for the Universities of Minnesota and Texas in the USA [114].

LEOPARD is caused by a missense mutation reducing protein tyrosine phosphatase activity in a protein coding area, called PTPN1, on chromosome 12 [115]. It can follow an autosomal dominant method of transmission or can be sporadic, and it is also characterized by high penetrance and genetic and phenotypic heterogeneity [112].

*Clinical manifestations.* LEOPARD syndrome includes multiple lentiginous lesions, ocular hypertelorism, pulmonary stenosis symptoms, abnormalities in genitalia, retarded growth, and sensorineural deafness [114]. These are the most characteristic features and they start presenting in childhood. Pectus excavatum, pectus carinatum, and a wide chest with a large distance between the nipples are very common findings. As far as the genitourinary tract is concerned, cryptorchidism, hypospadias, and delayed puberty are often found. Lentigines are brown-black macules which are usually seen in the chest, back, face and arms but not in the mucosa. They usually appear after or along with hypertrophic cardiomyopathy. Mitral valves prolapse and atrial and atrioventricular septal defects may also be seen, but hypertrophic cardiomyopathy is one of the most important and serious comorbidities [112].

**Figure 13:**
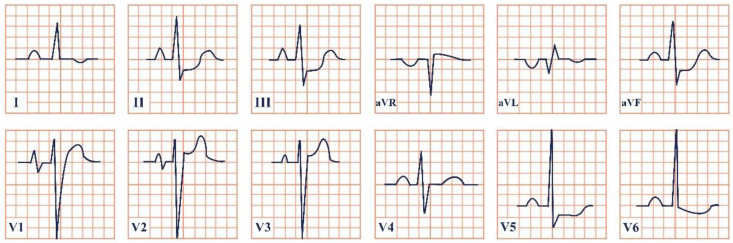
Electrocardiogram aspect in LEOPARD syndrome.

*Paraclinical diagnosis*. On the electrocardiogram, left or biventricular hypertrophy, repolarization abnormalities, prolonged QT-interval, and atrioventricular blocks may be seen (Figure 13). Echocardiography is required to visualize valvulopathies and assess the hypertrophy. Patients should undergo 24 h Holter ECG monitoring and cardiac stress testing for the detection of arrhythmias [116]. Genetic analysis is recommended and family member screening as well [115]. Prenatally, LEOPARD can be suspected if the fetus has hypertrophic cardiomyopathy and can be confirmed with the genetic testing of the amniotic fluid or chorionic villi [112].

*Diagnostic criteria.* The diagnosis is confirmed according to the following criteria: (1) Multiple lentigines, with at least three of the following: structural cardiac defects, electrocardiographic cardiac abnormalities, genitourinary abnormalities, neurological abnormalities, craniofacial deformities, growth disorders, and skeletal malformations. (2) If lentigines are absent, look for at least three of the characteristics mentioned above in the patient or close family [116,117].

*Prognosis*. Patients with no cardiac abnormalities or mild ones have a good prognosis overall but should be monitored annually. Patients who suffer from severe valvulopathies or hypertrophic cardiomyopathy are at high risk for arrhythmias and sudden cardiac death. Valvulopathies should be treated surgically with a valvulotomy or valvulectomy, and hypertrophic cardiomyopathy can be treated pharmacologically with myotomy-myectomy septal alcohol ablation or pacemaker implantation, depending on its properties and complications [112,118].

## 15. Conclusions

The electrocardiogram is a widely available, affordable, and non-invasive modality with a high diagnostic sensitivity. As modern cardiology evolves, so do the types of ECG and the settings where it is available, with portable 24 h Holter monitors, treadmill testing, and emergency electrocardiograms in the field. With cardiovascular disease being the leading cause of death worldwide, it is vital that healthcare professionals from all specialties have a thorough training on electrocardiogram interpretation, being able to identify not only common ECG findings but also rarer and more complicated ones. In this article, we provide information regarding the discovery of various ECG syndromes, their clinical manifestations and paraclinical investigations to be used, as well as the prognosis and treatment.

The diagnosis of the above-mentioned syndromes can be challenging for the physician, resulting in misdiagnosis and delay in treatment. The knowledge and understanding of these syndromes are critical for detecting early, treating appropriately, and preventing fatal arrhythmias and sudden cardiac death. Through this review, we hope to spread awareness of all these syndromes and their particularities in order to promote prompt recognition, referral, and improved survival rates.

## Figures and Tables

**Table 1:** Electrocardiogram syndromes.

ECG Syndrome	ECG Diagnostic Criteria	ECG Aspect
Wellen’s syndrome	– Normal ECG in angina, T wave changes in V2-3 when pain-free. – Absence of pathological Q waves. – No R wave loss.	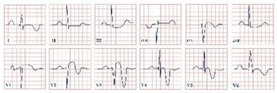
De Winter syndrome	– No ST-segment elevation in precordial leads. – Up-sloping ST-segment depression at J-point >1 mm. – Deep symmetrical T waves in precordial leads. – ST-segment elevation in aVR <0.5 mm. – May be preceded or followed by ST-segment elevation.	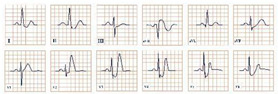
Kounis syndrome	– Evidence of myocardial ischemia on ECG in presence of systemic allergic reaction.	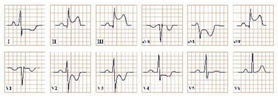
Takotsubo syndrome	– New and reversible ECG changes (ST-segment elevation, ST-segment depression, left bundle branch block, T-wave inversion, and/or QTc prolongation) during the acute phase (3 months).	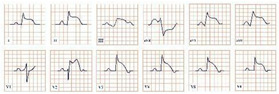
Yamaguchi syndrome	– Giant negative T waves (>10 mm), left ventricular hypertrophy and possibly signs of complications, including atrial fibrillation and ACS with ST-segment elevation.	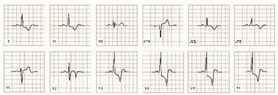
Wolff–Parkinson–White syndrome	– Short PR interval <120 ms. – Slurred upstroke at QRS beginning, called delta wave. – Wide QRS complex >120 ms. – T wave, ST-segment changes.	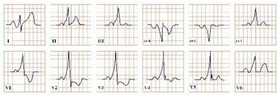
Lown–Ganong–Levine syndrome	– Short PR interval <120 ms. – Normal QRS complex <120 ms. – Absence of delta wave.	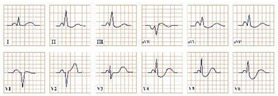
Brugada syndrome	– Type 1 consists of coved ST-segment elevations greater than 2 mm followed by an inverted T wave. – Type 2 consists of a saddle-back ST-segment elevation greater than 2 mm followed by a normal or biphasic T wave. – Type 3 is characterized by a coved or saddleback-shaped ST-segment elevation that is equal or less than 1 mm.	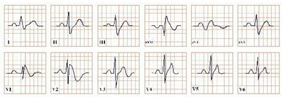
Frederick’s syndrome	– Atrial fibrillation “f waves” or atrial flutter waves. – Third degree AV block.	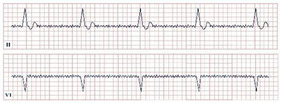
PAFIYAMA syndrome	– Onset as paroxysmal atrial fibrillation. – ST-segment elevation at J-point >0.1 mm in 2 leads. – Inverted T waves in 2 leads. – Left atrial enlargement signs. – Left ventricular hypertrophy signs.	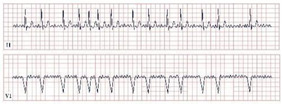
Haïssaguerre syndrome	– J-point elevation >1 mm in >2 contiguous inferior and/or lateral leads in a patient with history of resuscitation from otherwise unexplained ventricular fibrillation or polymorphic ventricular tachycardia. Presence of J-point elevation >1 mm in >2 contiguous inferior and/or lateral leads on ECG.	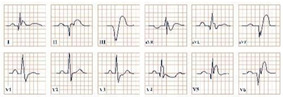
Osborn syndrome	– Haïssaguerre and Brugada syndrome criteria may be used.	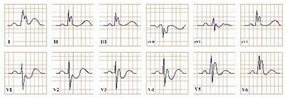
LEOPARD syndrome	– Left or biventricular hypertrophy. – Repolarization abnormalities. – Prolonged QT-interval. – Atrioventricular blocks.	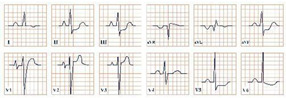

## Data Availability

Not applicable.
